# Use of a highly-sensitive cardiac troponin I assay in a screening population for hypertrophic cardiomyopathy: a case-referent study

**DOI:** 10.1186/1471-2261-13-70

**Published:** 2013-09-11

**Authors:** Catherine M McGorrian, Sarah Lyster, Andrew Roy, Heloise Tarrant, Mary Codd, Peter Doran, Maria Fitzgibbon, Joseph Galvin, Niall G Mahon

**Affiliations:** 1 Department of Cardiology, Mater Misericordiae University Hospital, Eccles St., Dublin 7, Ireland; 2 UCD School of Public Health, Physiotherapy and Population Science, UCD Belfield, Dublin 4, Ireland; 3 UCD School of Biomolecular and Biomedical Science, UCD, Belfield, Dublin 4, Ireland; 4 Department of Clinical Biochemistry, Mater Misericordiae University Hospital, Eccles St., Dublin 7, Ireland; 5 Clinical Research Centre, Mater Misericordiae University Hospital, Eccles St., Dublin 7, Ireland; 6 Department of Cardiology, Connolly Hospital Blanchardstown, Dublin 15, Ireland

**Keywords:** Hypertrophic cardiomyopathy, High-risk screening, Troponin I, Biomarkers, Highly-sensitive assays

## Abstract

**Background:**

Hypertrophic cardiomyopathy (HCM) is a genetic condition, and relatives of affected persons may be at risk. Cardiac troponin biomarkers have previously been shown to be elevated in HCM. This study examines the new highly-sensitive cardiac troponin I (hsTnI) assay in a HCM screening population.

**Methods:**

Nested case–control study of consecutive HCM sufferers and their relatives recruited from May 2010 to September 2011. After informed consent, participants provided venous blood samples and clinical and echocardiographic features were recorded. Associations between the natural log (ln) of the contemporary troponin I (cTnI) and hsTnI assays and markers of cardiac hypertrophy were examined. Multiple regression models were fitted to examine the predictive ability of hsTnI for borderline or definite HCM.

**Results:**

Of 107 patients, 24 had borderline and 19 had definite changes of HCM. Both TnI assays showed significant, positive correlations with measures of cardiac muscle mass. After age and sex adjustment, the area under the receiver operator characteristic (AUROC) curve for the outcome of HCM was 0.78, 95% CI [0.65, 0.90], for ln(hsTnI), and 0.66, 95% CI [0.51, 0.82], for ln(cTnI) (p=0.11). Including the hsTnI assay in a multiple-adjusted “screening” model for HCM resulted in a non-significant improvement in both the AUROC and integrated discrimination index.

**Conclusions:**

Both cTnI and hsTnI show a graded, positive association with measures of cardiac muscle mass in persons at risk of HCM. Further studies will be required to evaluate the utility of these assays in ECG- and symptom-based identification of HCM in at-risk families.

## Background

Hypertrophic cardiomyopathy (HCM) is a genetic condition with otherwise unexplained left ventricular hypertrophy in the absence of LV chamber dilatation [1]. It is a relatively common condition [2], and carries an increased risk of arrhythmia and sudden cardiac death, even in those persons with clinically “silent” or unrecognised HCM. Therefore, cardiac screening of first-degree relatives of HCM probands is now advised [3]. Cascade screening may be clinical with an electrocardiogram (ECG) and transthoracic echocardiogram (echo), genetic (if the family mutation is identified ), or a combination of both. However, such screening presents multiple challenges. For genetic evaluation, causative gene identification is currently possible in 40-70% of tested families [4]. In clinical evaluations, it can be a challenge to differentiate between early HCM changes and other findings such as the athlete’s heart [5] and hypertensive heart disease [6].

Biomarkers are naturally occuring molecules which may indicate a disease process. Cardiac troponins are biomarkers which are released due to myocyte necrosis, and cardiac troponin I (TnI) is specific to cardiac muscle. There is a known association between cardiac troponin levels and cardiac muscle hypertrophy [7-9]. Recently, high-sensitivity cardiac troponin I (hs cTnI) assays have become available [10], and will ultimately replace contemporary cTnI assays. Such hsTnI assays not only provide greater precision at the 99th percentile limits, but can also detect lower levels of troponin “leak” with greater sensitivity. It is not known how hsTnI assays may behave in persons with or at risk of HCM, but it may be that these lower levels of cTnI may provide useful screening information in such persons.

In this study, we aimed to examine the distribution of TnI, measured with both a standard contemporary (cTnI) and new highly-sensitive assay, in a high-risk population for HCM. A “proof of concept” investigation was undertaken to examine the association of cTnI and hsTnI with echocardiographic markers of HCM, and the incremental screening value of adding highly-sensitive biomarker data to ECG and clinical data was assessed.

## Methods

### Study population

The Family Heart Screening Study is a prospective single-centre cohort study of patients at risk of familial cardiac conditions, based in a screening clinic for familial cardiomyopathies and channelopathies. From March 2010 to August 2011, all patients attending for HCM screening were considered for study inclusion. Patients were included if they had a first or second degree relative with HCM, and were aged 18 and over. Only patients who provided informed consent for both data collection and serum sampling were included in this analysis. Ethical approval for this study was granted by the Mater Misericordiae University Hospital Research Ethics Committee, and the study was conducted with due regard to the principles of the Declaration of Helsinki.

Patients underwent protocol-driven clinical screening, with clinical history-taking and examination, pedigree analysis, and ECG and echo. Data were collected on baseline symptoms including chest pain, dyspnoea, palpitations and syncope. Patients were deemed to be symptomatic if they described any of these symptoms. ECGs were defined as abnormal if any typical “Group 2” ECG changes were noted (i.e. presence of ST depression or T-wave inversion, pathological Q waves, interventricular conduction delays, deviations in cardiac axis, long or short QT and/or Brugada-like repolarisation changes) [11]. Body surface area (BSA) was calculated for all persons in whom weight and height were available.

### Echocardiograph measurements

Transthoracic echo studies were performed by a senior echocardiographer using a commercially available system (Vivid 7, GE Healthcare, Horten, Norway) with a 3.5-MHz transducer. Images were obtained in standard views and were digitally stored for offline analysis (Echopac Version 7.0., GE Healthcare). The ejection fraction (EF) was calculated using Simpson’s rule [12]. Peak E and A velocities, E/A ratio and deceleration time (DT) were recorded using pulse wave (PW) Doppler in the apical 4-chamber view. Tissue Doppler PW analysis was also used to measure mitral annular velocities, with the sample volume at the septal and lateral annulus insertion of the mitral valve leaflets (septal and lateral S’, E’ and A’). Left ventricular mass (LV mass) was calculated using the American Society of Echocardiography (ASE) method [13]. All echo images were read offline by two observers blinded to patient history.

The interventricular septum diameter in diastole (IVSd) and left ventricular posterior wall diameter in diastole (LVPWd) were used to calculate the IVSd/LVPWd ratio. The relative wall thickness (RWT) was calculated by dividing the IVSd/LVPWd ratio by the left ventricular end-diastolic diameter (LVEDD) [12]. The maximal wall thickness (MWT) was calculated from the parasternal short-axis view in 2D Mode by taking the maximal thickness of the left ventricular wall in end diastole in any of the following segments: anterior septum, posterior septum, posterolateral wall and anterolateral wall. The Maron-Spirito index was calculated by taking the sum of the left ventricular (LV) maximal thickness at each of these segments, measured both at basal (mitral valve) and mid ventricular (papillary muscle) levels [14]. The “adjusted 2D-LVH”scale was calculated as described by Forissier et al., where 2D LVH score= 18.95+ (0.12*age in years) + (2.64*male sex) + (6.41*BSA in kg/m^2^) [15]. The echo- and tissue doppler-based risk score of Gandjbakhch et al. for HCM mutation carrier probability was calculated using the equation p = −19.1861 + (6.195 ×IVS/LPW) + (22.538 × RWT) + (0.5613 × septal E/Ea) [16].

### Biomarker analyses

A single lithium-heparin venous sample (8ml) was drawn and transferred to a refrigerator at 4°C. The samples were centrifuged within 4 hours of collection, and the plasma sample was assigned a unique identifier number and frozen directly at −80°C. The hsTnI analysis was performed using the ARCHITECT STAT High Sensitive Troponin I assay on the ARCHITECT i1000_SR_ system (Abbott Diagnostics). The concentration of the hsTnI was read relative to a standard curve derived with calibrators of known hsTnI concentration. The cTnI analysis was performed on samples which had undergone a single previous thaw, with an ARCHITECT STAT Troponin-I assay, also on the ARCHITECT i1000_SR_ system. The concentration of the cTnI present was read relative to a standard curve derived with calibrators of known cTnI concentration. Standard procedures were followed for callibration and norms.

For this study, a population group of 109 unaffected relatives (with a normal cardiovascular examination, ECG and echo) served to define the 99th centile for the normal population (in this study, 24.88 pg/ml). In healthy individuals, values of troponin (Tn) are low and many fall below the detection limit of contemporary assays. Therefore the 99th centile of the healthy population is recommended as a clinical decision cut off value. For the contemporary cTnI assay, the assay precision is ≤10% total coefficient of variation (CV) for samples ≥ 0.2 ng/mL with an analytical sensitivity of ≤ 0.01 ng/mL at the 95% confidence interval (CI). The hsTnI assay has a 10% CV at 0.047 ng/ml with a limit of detection ≤0.002 ng/ml. The analytical specificity is ≤0.1% cross-reactivity with skeletal troponin-I and ≤ 1% with cardiac troponin-T and troponin-C.

### Statistical analysis

Patient outcomes were defined using established echo criteria for HCM (1,3). Patients with a MWT <13 mm were deemed to have no evidence of HCM, with patients with a MWT of ≥15 mm deemed to have a definite finding of HCM. Patients with a MWT of 13-14 mm were deemed to have borderline changes. Descriptive statistics were used to describe the clinical and echo characteristics of the screened population by screening outcome, using contingency tables and the Pearson chi-square test to compare categorical variables and simple analysis of variance models (ANOVA) to compare continuous variables between the three groups. The distribution of the cTnI and hs cTnI biomarkers in the populations were examined, and a natural log transformation was used to allow the use of parametric statistics.

Pearson’s correlation coefficient was used to examine correlations between the biomarker measures and key echo measurements, and two way scatterplots with fitted ordinary least-squares regression lines were used. Analysis of the biomarkers proceded as suggested by Hlatky et al. [17]. Simple logistic regression models were fitted, adjusting for age and sex, with either cTnI or hsTnI as the independent variable, and the presence of an abnormal echo screening evaluation as the dependent variable. Area under the receiver operator characteristic (AUROC) curve was calculated and compared for the logistic models described [18]. To examine the potential utility of the biomarker in a clinic or screening context, three forward stepped logistic models were fitted, with the a possible or definite HCM finding on echo as the dependent variable. The first “simple” model had age, sex and symptoms (any of chest pain, dyspnoea, palpitations and syncope) as independent variables; the second “clinic model” then included abnormal or “group 2” ECG findings [11]; and the third “enhanced” model included ln(hsTnI). Measurement of the model r^2^, AUROC, and integrated discrimination index (IDI) was undertaken [19]. All analyses were performed with Intercooled Stata 11 (StataCorp™, Texas).

## Results

Complete clinical data were available on 107 patients who underwent screening for HCM. The baseline population characteristics are shown in Table 1. Eight patients were in fact the family proband or index case, who had been referred for assessment to confirm the HCM diagnosis. Of these probands, seven had definite and one had borderline HCM changes on echo. Table 2 describes the echo characteristics in this population. As might be expected by the echo-based stratificication method used, echo markers of increased left ventricular thickness and reduced diastolic function, as well as three echo-based scores of left ventricular hypertrophy and HCM [14-16], were observed to increase by diagnostic stratum.

**Table 1:** **Baseline characteristics of the study population, stratified by echocardiographic criteria of HCM**^**1,3**^

	**Total (n = 107)**	**Normal screening echo (n=64)**	**Borderline HCM (n=24)**	**Definite HCM (n=19)**	
***Demographic data***					
**Age in years: mean [SD]**	39.30 (13.86)	35.63 [12.26]	44.92 [12.793]	44.59 [16.63]	F=6.15, p=0.003
**Male sex: n (%)**	63 (58002E9%)	32 (50.0%)	20 (83.3%)	11 (57.9%)	Χ2=8.01, p=0.018
**BMI in kg/m**^**2**^**: mean [SD]**	26.94 (3.90)	25.67 (3.90)	29.08 [3.88]	27.70 [3.07]	F=5.97, p=0.004
**Relationship to the family proband**					
**Proband: n (%)**	8 (7.5%)	0 (0%)	1 (4.2%)	7 (36.85%)	Fishers exact p<0.005
**1**^**st**^**degree relative : n (%)**	82 (76.6%)	53 (82.8%)	20 (83.3%)	9 (47.4%)	
**2**^**nd**^**degree or higher : n (%)**	17 (15.9%)	11 (17.2%)	3 (12.5%)	3 (15.8%)	
***Clinical History***					
**Any symptoms: n (%)**	36 (33.6%)	18 (28.1%)	8 (33.3%)	10 (52.5%)	Χ2=3.94, p=0.139
**Chest pain: n (%)**	10 (9.4%)	7 (10.9%)	2 (8.3%)	1 (5.3%)	
**Dyspnoea: n(%)**	12 (11.2%)	5 (7.8%)	3 (12.5%)	4 (21.1%)	Fishers exact p=0.267
**Palpitations: n(%)**	17 (15.9%)	7 (10.9%)	4 (16.7%)	6 (31.6%)	Fishers exact p=0.089
**History of syncope: n(%)**	6 (5.6%)	2 (3.1%)	2 (8.3%)	2 (10.5%)	Fishers exact p=0.286

*BMI* Body Mass Index.

**Table 2:** **Echocardiographic characteristics of the study population, stratified by standard echo criteria for HCM**^**1,3**^

	**Normal screening echo (n = 64)**	**Borderline HCM (n = 24)**	**Definite HCM (n = 19)**	Test statistic*	**P-value**
***2D-TM Data: mean [SD]***					
**IVSd (mm)**	10.30 (2.17)	12.38 (2.36)	15.53 (4.50)	F= 27.33	<0.0001
**LVPWd (mm)**	9.22 (1.67)	11.00 (2.57)	11.32 (2.31)	F=11.79	<0.0001
**IVS/LVPW ratio**	1.13 (0.20)	1.17 (0.33)	1.38 (0.33)	F=6.77	0.0017
**LVIDd (mm)**	48.86 (5.76)	50.42 (6.00)	47.16 (7.92)	F= 1.45	0.239
**LA diameter (mm)**	34.75 (5.62)	38.98 (4.02)	36.72 (4.96)	F= 5.73	0.0044
**LVEF M Mode (%)**	61.81 (7.08)	66.17 (7.46)	68.33 (11.82)	F=5.67	0.0046
**LVOT gradient (mmHg)**	5.32 (1.87)	5.91 (2.39)	7.00 (3.27)	F=3.35	0.0399
**RWT (mm)**	0.38 (0.06)	0.44 (0.07)	0.50 (0.08)	F=26.78	<0.0001
**MWT (mm)**	10.62 (1.13)	13.42 (0.50)	17.63 (3.65)	F= 118.46	<0.0001
**Maximal LV wall dimensions in diastole:**					
**Antero- septal wall (mm)**	10.12 (1.37)	12.58 (1.10)	16.63 (3.39)	F=93.87	<0.0001
**Postero- septal wall (mm)**	9.88 (1.32)	12.67 (1.27)	15.84 (4.57)	F=55.64	<0.0001
**Antero-lateral wall (mm)**	9.22 (1.13)	10.83 (1.61)	11.53 (2.09)	F=23.74	<0.0001
**Posterior wall (mm)**	9.25 (1.09)	10.96 (1.08)	11.68 (2.03)	F=32.74	<0.0001
**LV Mass (g)**	172.55 (48.34)	230.29 (60.66)	268.53 (133.37)	F=14.96	<0.0001
***Pulsed Doppler***					
**Peak E velocity (cm/s)**	0.80 (0.17)	0.73 (0.22)	0.79 (0.14)	F=1.60	0.206
**Peak A velocity (cm/s)**	0.58 (0.17)	0.58 (0.13)	0.60 (0.18)	F=0.16	0.854
**E/A ratio**	1.49 (0.49)	1.31 (0.51)	1.45 (0.61)	F=0.98	0.379
**E deceleration time (ms)**	187.87 (60.29)	203.50 (33.91)	220.11 (61.30)	F=2.60	0.079
***Tissue Doppler***					
**Septal Sa (cm/s)**	8.25 (2.35)	7.68 (2.48)	7.10 (1.99)	F=1.54	0.220
**Septal Ea (cm/s)**	10.22 (3.33)	7.01 (3.29)	7.90 (3.90)	F=8.02	0.0006
**Septal Aa (cm/s)**	8.89 (2.11)	8.96 (3.58)	8.09 (2.64)	F=0.60	0.550
**Lateral Sa (cm/s)**	10.13 (3.38)	8.41 (3.14)	8.29 (2.44)	F=3.18	0.047
**Lateral Ea (cm/s)**	12.87 (5.99)	9.95 (4.03)	11.19 (4.27)	F=2.52	0.087
**Lateral Aa (cm/s)**	8.85 (2.99)	7.87 (3.39)	7.78 (3.50)	F=1.06	0.350
***Echocardiograph composite scores***					
**Adjusted 2D-LVH score**	36.28 (2.69)	39.71 (2.69)	39.03 (2.80)	F=12.33	<0.0001
**Spirito index (mm)**	38.47 (3.71)	47.04 (3.03)	55.68 (9.53)	F=90.81	<0.0001
**Gandjbakhch risk score**	−2.78 (1.59)	−0.95(3.77)	0.57 (2.59)	F=11.91	<0.0001

* Comparisons across the strata of echo findings were made using ANOVA.

*IVSd* Interventricular septum diameter in diastole.

*LVPWd* Left ventricular posterior wall diameter in diastole.

*LVIDd* Left ventricular internal diameter in diastole.

*LA* Left atrium.

*LVEF* Left ventricular ejection fraction.

*LVOT* Left ventricular outflow tract.

*RWT* Relative wall thickness.

*MWT* Maximal wall thickness.

**Figure 1:**
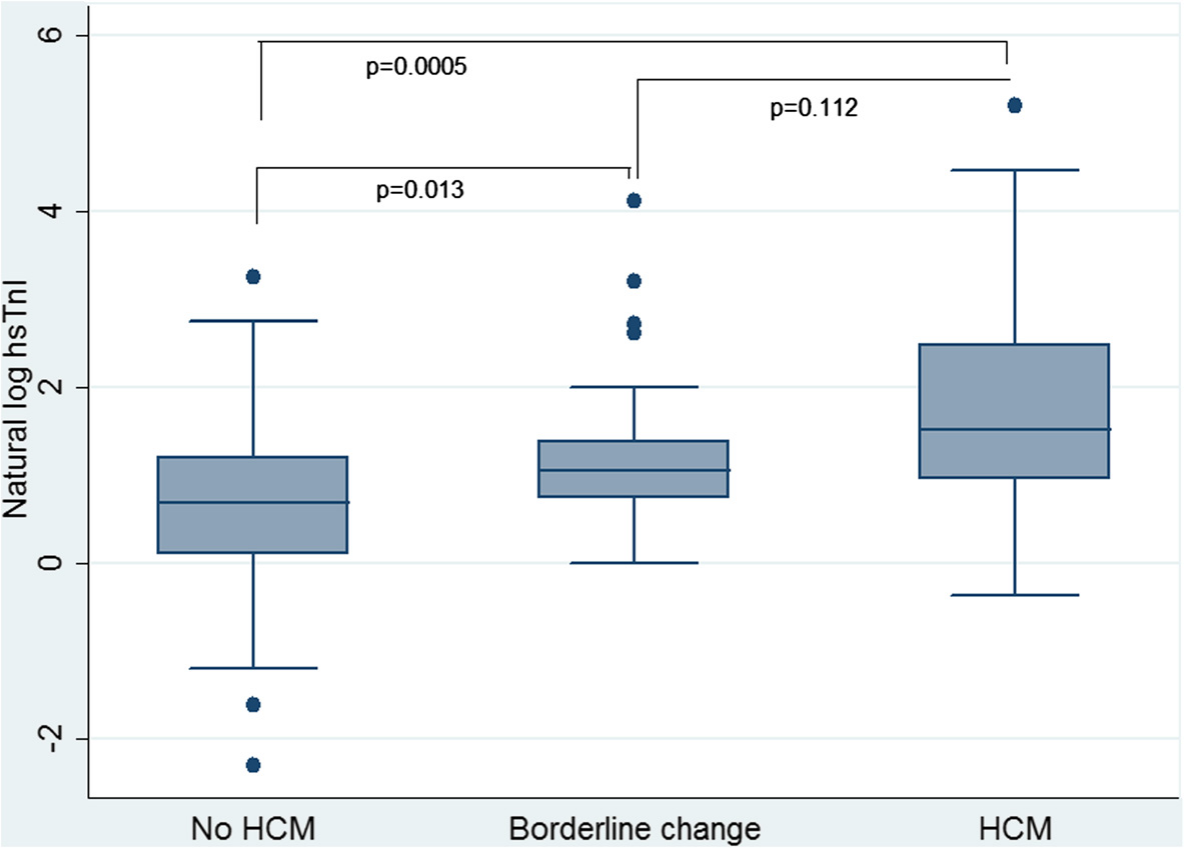
**Box plot of the natural log of highly-sensitive cardiac troponin I by HCM groups as stratified by standard echo criteria.** Footnote for Figure 1: p values shown are from the Wilcoxon rank sum test. For the the difference in ln(hsTnI) between the no HCM and Borderline change groups: z=−2.47, p=0.013. For the the difference in ln(hsTnI) between the Definite HCM and Borderline change groups: z=−1.59, p=0.112. For the the difference in ln(hsTnI) between the Definite HCM and no HCM groups: z=−3.468, p=0.0005.

The results of the cardiac troponin assays are shown in Table 3, by screening outcome. In total, 104 patients had valid results for the hsTnI assay, and 93 for the cTnI assay. Significant increases in TnI both by the contemporary and highly-sensitive assay were seen across the HCM strata. This graded association was also seen when 99th percentile cut points were applied. A box plot displaying this significant increase in ln(hsTnI) is shown in Figure 1. This relationship between both cTnI and hsTnI and echo-based measures of LV hypertrophy and diastolic function was examined further using Pearson’s correlation coefficient, and strong, positive relationship were again noted between the measures of LV hypertrophy and TnI (Additional file 1: Table S1).

**Table 3:** Description of the cardiac troponin and highly sensitive troponin findings in the screening population

		**Normal screening echo**	**Borderline HCM**	**Definite HCM**	**Test statistic; P-value**
**Sample size:**		56	20	17	
**Cardiac Troponin I**	Log cTnI: mean(SD)	−4.71 (0.01)	−4.60 (0.34)	−4.31 (0.94)	F= 5.63; p= 0.005
	Abnormal cTnI: n(%)	0 (0%)	1 (5%)	3 (18.8%)	Fisher’s exact p= 0.006
**Sample size:**		63	22	19	
**High-sensitivity cardiac troponin I**	Log hsTnI: mean (SD)	0.62 (1.08)	1.34 (1.00)	1.94 (1.45)	F=11.06; p<0.0001
	Abnormal hsTnI: n(%)	1 (1.6%)	1 (4.6%)	4 (22.2%)	Fisher’s exact p=0.008

*HCM* Hypertrophic cardiomyopathy.

**Figure 2:**
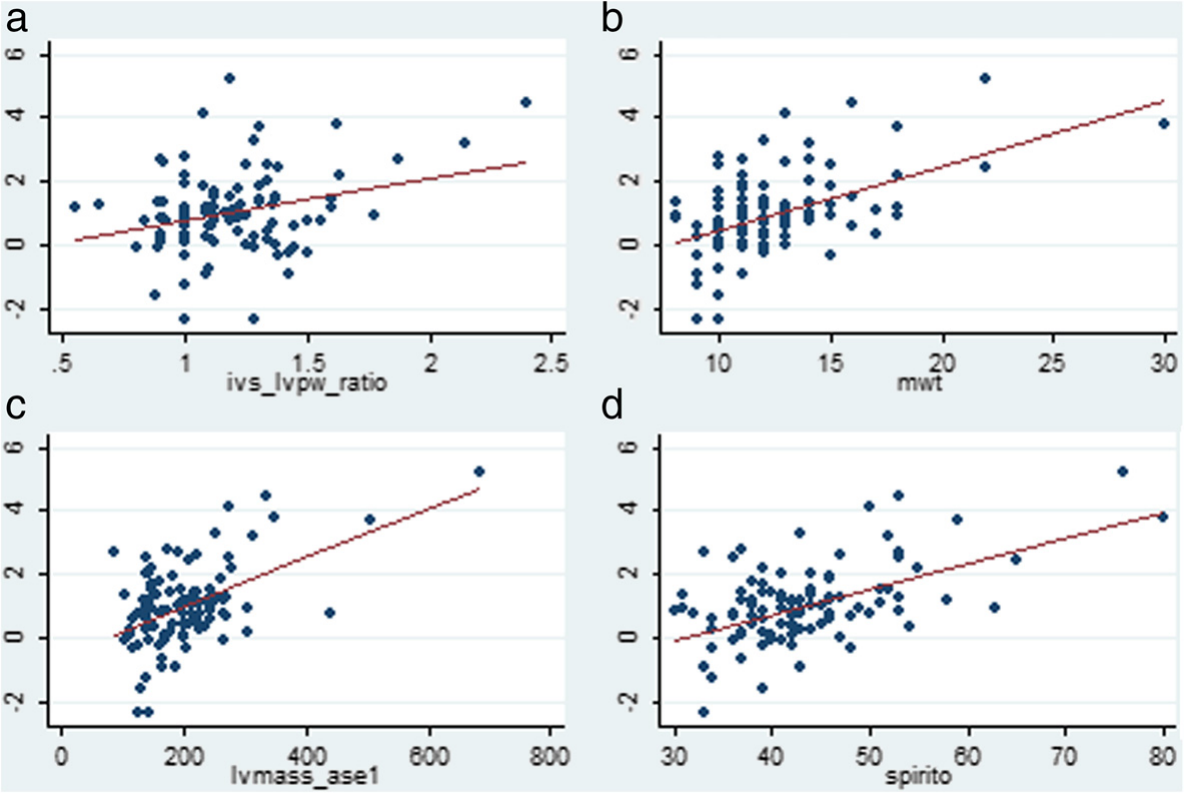
**Scatterplot with fitted linear regression line, showing the association between the natural log of highly sensitive cardiac troonin I with a. the IVS:LVPW ratio, b. the MWT, c. the LV mass estimate and d. the Spirito index.** Footnote for Figure 2**:** Regression coefficient for the IVSd: LVPWd ratio was 0.008, 95% CI [0.52, 2.19], p=0.002, adjusted R^2^ 0.08. Regression coefficient for the LV maximal wall thickness was 0.20, 95% CI [0.13, 0.26], p<0.0005, adjusted R^2^ 0.25. Regression coefficient for the LV mass was 0.088, 95% CI [0.005, 0.010], p<0.0005, adjusted R^2^ 0.26. Regression coefficient for the Spirito score was 0.08, 95% CI [0.06, 0.10], p<0.0005, adjusted R^2^ 0.29.

Figure 2 shows the association between the natural log of hsTnI and key measures of LV mass (MWT, IVS:LVPWd ratio, LV mass and the Spirito score). Ordinary least-squares regression lines were fitted, with adjusted R^2^ values of up to 0.29 for the model with the Spirito score as the dependent variable. Simple and multiple-adjusted logistic models for possible or definite HCM by echo criteria [1,3] are shown in Table 4. For both models, the odds ratio associated with hsTnI, but not cTnI, was statistically significant. Furthermore, the models which included hsTnI had non-significant increases in AUROC when compared with equivalent models with the cTnI assay as a covariable.

**Table 4:** Association of both standard and highly sensitive cardiac troponin I assays with an echo diagnosis of definite HCM, using logistic regression analysis

		**Odds ratio (95% CI)**	**P value**	**Pseudo R2**	**AUROC (95% CI)**
***cTnI models***					
Model 1*	Natural log of cTnI	4.20 (0.94, 18.80)	0.060	0.102	0.66 (0.51, 0.82)
Model 2 **		2.56 (0.54, 12.14)	0.236	0.197	0.76 (0.62, 0.91)
***hsTnI models***					
Model 1*	Natural log of hsTnI	2.35 (1.39, 3.96)	0.001	0.173	0.78 (0.65, 0.90)
Model 2 **		1.82 (1.02, 3.24)	0.042	0.231	0.81 (0.70, 0.93)

* Model 1 adjusted for age and sex.

** Model 2 adjusted for age, sex, presence of group 2 ECG abnormalities, and symptoms of dyspnoea, chest pain, syncope or palpitations.

Footnote: For the ln(cTnI): comparison between AUROC in Model 1 vs Model 2: χ2=1.90, p=0.168. For the ln(hsTnI): comparison between AUROC in Model 1 vs Model 2: χ2=1.03, p=0.310. For the comparison between the AUROCs in Model 2 for the models with ln(cTnI) and ln(hsTnI): χ2= 0.87, p=0.351.

We examined the potential screening utility of the hsTnI assay in a screening setting for HCM. Table 5 shows incremental though statistically non-significant gains in model R^2^and AUROC when the hsTnI assay was added to the “screening” model, and there was a trend towards an improvement in IDI with the “enhanced screening” model when compared to the model without ln(hsTnI). The sensitivity of the ln(hsTnI) “enhanced screening” model was 53.7% and specificity was 88.9%, with a positive predictive value of 75.9% and a negative predictive value of 74.7%.

**Table 5:** HCM screening and the effect of the addition of the troponin measures screening models, using logistic regression with boderline or definite HCM findings at echo as the dependent variable

**Model**	**Covariables**	**n**	**Pseudo R2**	**AUROC (95% CI)**
**Simple model**	Age, sex and symptoms	104	0.177	0.75 (0.65, 0.86)
**Screening model**	Age, sex, symptoms and “Group 2” ECG changes	104	0.189	0.77 (0.67, 0.87)
**Enhanced screening model with cTnI**	Age, sex, symptoms, “Group 2” ECG changes and ln(cTnI)	104	0.201	0.75 (0.63, 0.86)
**Enhanced screening model with hsTnI**	Age, sex, symptoms, “Group 2” ECG changes and ln(hsTnI)	104	0.225	0.78 (0.69, 0.88)

Footnote: Integrated discrimination index (IDI) for the addition of the ln(cTnI) to the “Screening” model is 0.025 (standard error 0.024), p=0.299. Integrated discrimination index (IDI) for the addition of the ln(hsTnI) to the “Screening” model is 0.036 (standard error 0.019), p=0.056.

## Discussion

### Description of key findings

This study aimed to examine the association between cardiac troponin I and the clinical diagnosis of hypertrophic cardiomyopathy in a high-risk screening population, with a focus on the new highly-sensitive cTnI assay method. There was a clear and consistent graded association between cTnI (measured both by the contemporary and highly-sensitive assays) and measures of LV hypertrophy, whereas the association of the troponin measurements with functional measures such as diastolic function were less clear. While improvements in IDI and AUROC were noted when the hsTnI assay measure was added to a clinic-based regression model to predict the finding of an echo finding consistent with a clinical diagnosis of borderline or definite HCM, these improvements were not statistically significant.

### Where this fits in the literature

There is emerging interest in the possibility of using highly-sensitive troponin measures in risk stratification in cardiovascular diseases. Highly-sensitive troponin I has been shown to contribute to athersclerotic CVD and heart failure risk in primary prevention populations, even after adjustment for multiple traditional risk factors [20,21]. However, it is not clear why troponins I and T may be elevated in HCM. Current theories include the concept that the elevation may be due to myocyte necrosis from a mismatch between the hypertrophied myocardium and a compromised coronary blood supply, or that the elevation is caused by the underlying genetic abnormality [22].

Cardiac troponin assays have been previously established to be associated with degree of hypertrophy in patients with known HCM [23]. CTnI is correlated with maximal LV wall thickness in patients with hypertrophic cardiomyopathy [8]. Moreno et al. described an outpatient population with HCM, in who 42% had an elevated hsTnT level, and patients with higher hsTnT levels were more likely to have symptoms of dyspnoea and/or fibrosis on cardiac MRI evaluation [9]. It has also been reported to be elevated in HCM caused by Fabry’s disease [24]. Cardiac troponin I has also been linked to outcome status in HCM, with a combination of cTnI and BNP predicting adverse cardiovascular outcomes [25]. However, the utility of cTnI and hsTnI in a HCM screening population has not previously been evaluated. Furthermore, we are not aware of any previous study which has compared the relative utility of the two assay types in such a population.

### Strengths and limitations

This study reports the first examination of a new highly sensitive TnI assay in patients both with, and at risk of, HCM. Furthermore, our analysis presents a novel potential use for cTnI and hsTnI, using a high-risk screening population for HCM. The population was well phenotyped, with echos read independently and in a blinded manner. Our study has some limitations. The data described are cross-sectional data, and clinical outcome data are not yet available. A cohort study on this population is ongoing. Other authors have used an analysis endpoint of HCM genotype status. This was not consistently available for our patients, and we note that HCM genotype may not be available in many clinical situations where a rapid decision on risk status is required. An echocardiographic end-point has good face validity and a well-established clinical application. Tissue and pulsed Doppler measures of diastolic dysfunction were used in this study, and were seen not to have a consistent relationship with cTnI or hsTnI. Use of a further robust measure such as speckle-tracking echocardiography should however be considered for future studies. The sample size was small, and therefore this study aimed to establish “proof of concept” only [17]. Whilst small improvements in “clinic” stratification were seen using hsTnI, these were not statistically significant. Further study recruitment is underway. The study sample are from a high-risk screening population, and it is not known how hsTnI may add to HCM diagnosis in population-screening samples.

### Implications for practice

This study shows a potential role for cardiac troponin assays, in particular assays of hsTnI, in HCM screening. This finding needs to be replicated in other studies and also ideally examined in a prospective cohort setting. We examined a “high-risk” screening population for HCM. Population screening for inherited cardiac diseases is a topic of much debate, and there is particular focus on screening young athletes, in whom both a questionnaire and ECG are recommended [26]. However, identification of HCM and risk stratification can be clinically challenging. Furthermore, in young patients and sportspersons in particular, the ramifications of a HCM diagnosis can be substantial [27]. Highly-sensitive troponin assays are relatively inexpensive, and will be widely available in the future. This study provides a rationale for further investigation of the utility of this measure in the identification and management of patients with HCM.

## Conclusions

This is the first study to examine a new hsTnI assay in persons at risk of HCM. Both cTnI and hsTnI are shown to have a graded, positive association with measures of muscle mass in persons with and at risk of HCM. There was a non-significant increase in AUROC with the addition of hsTnI to the clinic “screening” model. The cTnI and new hsTnI assay may add to ECG- and symptom-based identification of HCM in at-risk families, although further larger scale studies will be required to evaluate this.

## Competing interests

The authors declare that they have no competing interests.

## Authors’ contributions

CMcG, AR, MC, PD, MF, JG and NM designed the study. CMcG, AR and SL collected the data. CMcG, SL, HT and MF performed the TnI analyses. CMcG and SL performed the data analysis and wrote the manuscript. All co authors provided comments on the manuscript. All authors read and approved the final manuscript.

## Pre-publication history

The pre-publication history for this paper can be accessed here:

http://www.biomedcentral.com/1471-2261/13/70/prepub

## Supplementary Material

Additional file 1: Table S1 Pair-wise correlation between the cardiac troponin I measurements and selected key echocardiographic features and scores. Click here for file
