# Supplementary material for: Use of a highly-sensitive cardiac troponin I assay in a screening population for hypertrophic cardiomyopathy: a case-referent study
Source: BMC Cardiovasc Disord. 2013 Sep 11;13:70. doi: 10.1186/1471-2261-13-70 (PMC3849957; doi:10.1186/1471-2261-13-70)
Supplement: Additional file 1: Table S1 — Pair-wise correlation between the cardiac troponin I measurements and selected key echocardiographic features and scores. [file 1471-2261-13-70-S1.doc]

**Supplementary Table: Pair-wise correlation between the cardiac troponin I measurements and selected key echocardiographic features and scores**

| **Echo feature** | **Cardiac troponin I (natural log)** | | **Highly sensitive Cardiac troponin I (natural log)** | |
| --- | --- | --- | --- | --- |
|  | **Pearson’s correlation coefficient** | **P value** | **Pearson’s correlation coefficient** | **P value** |
| IVSd | 0.509 | <0.001 | 0.466 | <0.001 |
| IVSd: LVPWd ratio | 0.315 | 0.0020 | 0.302 | 0.002 |
| LVIDd | 0.141 | 0.150 | −0.148 | 0.167 |
| LA diameter | 0.233 | 0.020 | 0.012 | 0.911 |
| LVOT gradient | 0.061 | 0.577 | −0.047 | 0.677 |
| Ejection fraction by M Mode | 0.053 | 0.618 | 0.075 | 0.454 |
| Relative wall thickness | 0.211 | 0.0420 | 0.267 | 0.006 |
| Maximal wall thickness | 0.418 | <0.001 | 0.511 | <0.001 |
| LV mass | 0.655 | <0.001 | 0.515 | <0.001 |
| Septal E/Ea ratio | 0.071 | 0.540 | 0.196 | 0.072 |
| Mitral E/A ratio | −0.016 | 0.884 | −0.007 | 0.94 |
| Adjusted 2D LVH (Forrisier) score (15) | −0.001 | 0.995 | 0.155 | 0.196 |
| Spirito index (14) | 0.485 | <0.001 | 0.543 | <0.001 |
| Gandjbakhch risk score (16) | 0.222 | 0.053 | 0.368 | 0.0005 |
| Cardiac troponin I (natural log) | - | - | 0.603 | <0.001 |

IVSd *Interventricular septum diameter in diastole*

LVPWd *Left ventricular posterior wall diameter in diastole*

LVIDd *Left ventricular internal diameter in diastole*

LA *Left atrium*

LVOT *Left ventricular outflow tract*
